# The efficacy and safety of mivacurium in pediatric patients

**DOI:** 10.1186/s12871-017-0350-2

**Published:** 2017-04-17

**Authors:** Ruifeng Zeng, Xiulan Liu, Jing Zhang, Ning Yin, Jian Fei, Shan Zhong, Zhiyong Hu, Miaofeng Hu, Mazhong Zhang, Bo Li, Jun Li, Qingquan Lian, Wangning ShangGuan

**Affiliations:** 1 Department of Anesthesiology, Critical Care and Pain Medicine, The Second Affiliated Hospital and Yuying Children’s Hospital of WenZhou Medical University, 109 West Xueyuan Road, Wenzhou, 325027 China; 2 Department of Anesthesiology, Nanjing Children’s Hospital, Nanjing, 210008 China; 3 Department of Anesthesiology, The Children’s Hospital of Zhejiang University School of Medicine, Hangzhou, 310003 China; 4 Department of Anesthesiology, Shanghai Jiao Tong University School of Medicine-Affiliated Shanghai Children’s Medical Centre, Shanghai, 200127 China; 5 Present address: The Affiliated Hospital of Chengde Medical College, Chengde, 067000 China; 6 Present address: ZhongDa Hospital of Southeast University, Nanjing, 210009 China

**Keywords:** Mivacurium, Children, Neuromuscular block, Histamine, Efficacy

## Abstract

**Background:**

Mivacurium is the shortest acting nondepolarizing muscle relaxant currently available; however, the effect of different dosages and injection times of intravenous mivacurium administration in children of different ages has rarely been reported. This study was aimed to evaluate the muscle relaxant effects and safety of different mivacurium dosages administered over different injection times in pediatric patients.

**Methods:**

Six hundred forty cases of pediatric patients, aged 2 m-14 years, ASA I or II, were divided into four groups (Groups A, B, C, D) according to the age class (2–12 m, 13–35 m, 3–6 years and 7–14 years) respectively, also each group were divided into four subgroups by induction dose (0.15, 0.2 mg/kg in 2–12 m age class; 0.2, 0.25 mg/kg in other three age classes), and mivacurium injection time (20 s, 40 s), totally 16 subgroups. Neuromuscular transmission was monitored with supramaximal train-of-four stimulation of the ulnar nerve. Radial artery blood (1 ml) was sampled to quantify plasma histamine concentrations before and 1, 4, and 7 min after mivacurium injection (P0, P1, P2 and P3).

**Results:**

Five hundred sixty-two cases completed the study. There were no demographic differences within the four groups. The onset time of 0.2 mg/kg groups in 2–12 m aged patients were shorter than those of 0.15 mg/kg groups (189 ± 64 s vs. 220 ± 73 s, 181 ± 60 s vs. 213 ± 71 s, *P* <0.05), and the recovery times were no statistical differences. The T1 25% recovery time of 0.2 mg/kg in 3–6 years aged patients was shorter than that of 0.25 mg/kg group (693 ± 188 s vs. 800 ± 206 s, *P* <0.05). The onset and recovery times of mivacurium were not different in 13–35 m and 7–14 years aged patients. The plasma concentrations of histamine at P0, P1, P2 and P3 were not different within four groups.

**Conclusions:**

The induction dose and injection time of mivacurium had mostly insignificant effects on onset and recovery times. The main exception to this was that in 2–12 m aged patients, increasing the dose of mivacurium from 0.15 to 0.2 mg/kg accelerated the onset time by about 30 s. Mivacurium produced no significant release of histamine in any age group at the doses studied.

**Trial registration:**

ClinicalTrials.gov Identifier-NCT02117401, July 14, 2014. (Retrospectively registered)

## Background

Mivacurium is a short-acting non-depolarizing neuromuscular blocker with a combination of the bisbenzyltetrahydroisoquinolinium structure of atracurium and the enzymatically degradable ester linkage of suxamethonium, and is quickly hydrolyzed by plasma cholinesterase (pChe). Mivacurium has been approved for use in children, and its recovery time in children is about 30% faster than in adults [1]. In Germany, mivacurium is predominantly used for pediatric anesthesia [2]. Mivacurium has insignificant accumulation, which results in rapid spontaneous recovery from the neuromuscular blockade and does not significantly change cardiocirculatory parameters in pediatric patients [3, 4]. The use of reversal agent after administration of mivacurium in normal patients is not necessary due to the rapidity of spontaneous recovery [5]. In addition, mivacurium is considered to cause mild to moderate histamine release, but its release is less in children as compared to adults [6]. Because of its rapid onset, short duration of action, lack of accumulation, short recovery, and relative lack of adverse effects, mivacurium was chosen for cases of short duration and pediatric surgery.

However, the effect of different dosages and injection times of intravenous mivacurium administration in different aged children has rarely been reported. In this study, we hypothesized that different dosages and injection times of mivacurium as a bolus could have different effects on its pharmacodynamics and histamine release in different aged children.

## Methods

### Patient selection

A prospective randomized cohort study was conducted among four children’s hospitals. Ethical approval for this study was provided by the Ethics Committee of the Second Affiliated Hospital and Yuying Children’s Hospital of Wenzhou Medical University (Chief centre, Number 2011-04-2). After written informed consent from parents or guardians was obtained, 640 children, aged 2 months to 14 years, physical status ASA I or II, scheduled to undergo elective general anesthesia with tracheal intubation were enrolled in the study among four hospitals, 160 cases for each hospital. The planned surgical duration was from 30 to 120 min. The exclusion criteria included: body mass index < 13.5 kg/m^2^ or > 31 kg/m^2^; severe respiratory or cardiovascular system disease and hepatic or renal insufficiency; history of neuromuscular disorder; airway abnormalities; abnormal plasma cholinesterase activity or pseudo cholinesterase deficiency (pChe test is one of preoperative routine tests in our hospital); diabetes; serious acid–base imbalance or electrolyte disorder; muscle relaxant administration during the previous 7 days; mivacurium allergy; and participation in any clinical subjects within the previous 30 days before the study.

### Interventions

Randomization was stratified independently in each hospital by age class (2–12 months, 13–35 months, 3–6 years and 7–14 years), induction dose (0.15 mg/kg mivacurium and 0.2 mg/kg mivacurium in 2–12 months age class, 0.2 mg/kg mivacurium and 0.25 mg/kg mivacurium in other three age classes) and mivacurium injection time (20 s, 40 s). All patients were divided into 4 groups (Groups A, B, C, D) and 16 subgroups, summarized in Tables 1, 2, 3 and 4. Sealed envelopes containing patient data were randomly provided to each study institution. Study personnel responsible for data collection were unaware of which group each patient belonged to and were allowed to enter the operating room only after adequate sedation had been administered to each child.

**Table 1:** Demographic data of group A (≥2, <12 months; *n* = 155)

Subgroups	n	Percentage in group A (%)	Sex (M/F)	Body weight (kg)	Induction dose (mg/kg)	Injecting time (s)
Group 1	39	25	27/12	8.5 ± 2.4	0.15	20
Group 2	37	24	23/14	8.6 ± 2.2	0.15	40
Group 3	39	25	27/12	9.1 ± 2.4	0.20	20
Group 4	40	26	26/14	8.8 ± 2.0	0.20	40

There was no significant different for sex ratio and body weight among four subgroups

**Table 2:** Demographic data of group B (13–35 months, *n* = 106)

Subgroups	n	Percentage in group A (%)	Sex (M/F)	Body weight (kg)	Induction dose (mg/kg)	Injecting time (s)
Group 5	29	27	21/8	13.1 ± 6.1	0.20	20
Group 6	25	24	17/8	11.5 ± 2.1	0.20	40
Group 7	28	26	19/9	14.3 ± 7.2	0.25	20
Group 8	24	23	12/12	12.6 ± 3.1	0.25	40

There was no significant different for sex ratio and body weight among four subgroups

**Table 3:** Demographic data of group C (3–6 years, *n* = 141)

Subgroups	n	Percentage in group A (%)	Sex (M/F)	Body weight (kg)	Induction dose (mg/kg)	Injecting time (s)
Group 9	34	24	22/12	20.3 ± 5.2	0.20	20
Group 10	36	26	23/13	19.1 ± 5.9	0.20	40
Group 11	35	24	23/12	19.8 ± 6.5	0.25	20
Group 12	36	26	24/12	17.9 ± 5.5	0.25	40

There was no significant different for sex ratio and body weight among four subgroups

**Table 4:** Demographic data of group D (7–14 years, *n* = 160)

Subgroups	n	Percentage in group A (%)	Sex (M/F)	Body weight (kg)	Induction dose (mg/kg)	Injecting time (s)
Group 13	40	25	26/14	31.1 ± 11.3	0.20	20
Group 14	40	25	27/13	34.6 ± 17.2	0.20	40
Group 15	40	25	30/10	33.7 ± 11.8	0.25	20
Group 16	40	25	27/13	32.2 ± 13.1	0.25	40

There was no significant different for sex ratio and body weight among four subgroups

### Neuromuscular monitoring

Neuromuscular transmission was monitored with acceleromyography (TOF WATCH SX). The ulnar nerve was stimulated at the wrist by a train-of four (TOF) stimulation (2 Hz, 0.2 ms) every 12 s and neuromuscular function was measured at the adductor pollicis. The four fingers, excluding the thumb, were immobilized. Two surface stimulating electrodes were put over the ulnar nerve at the wrist 2 cm apart, the negative electrode distal to the positive one. The acceleration indicator was fixed to the volar aspect of the distal part of the thumb. The ulnar nerve was stimulated with 60 mA energy. Palmar skin temperature was measured. Muscle relaxation monitoring was started after induction of anesthesia but before the administration of the muscle relaxant. When the supramaximal stimulation was achieved and was stable for 5 min, mivacurium was administered to the patient. After tracheal intubation was performed, the stimulating pattern of muscle relaxation monitoring was changed to train-of-four (TOF) stimulation. When the first twitch (T_1_) recovered to 25%, an additional bolus dose of mivacurium was injected up to a total of 0.1 mg/kg. After full recovery of neuromuscular function (T_1_ ≥ 95%), the endotracheal tube was removed and the patient was discharged from the operating room to post-anesthesia care unit (PACU).

### Performance of anesthesia

Uncooperative children were premedicated with ketamine 2.0 mg/kg, midazolam 0.5 mg/kg, and atropine 0.02-0.03 mg/kg intramuscularly; cooperative children were also sedated with ketamine 1.0-2.0 mg/kg, midazolam 0.1-0.2 mg/kg, and atropine 0.02 mg/kg, after the intravenous catheterization in the operating room. A pulse oximeter finger probe was attached to the patient. Other standard monitors in the operating room consisted of heart rate (HR), electrocardiogram (ECG), pulse oximetry (SpO_2_), non-invasive blood pressure (BP), and capnometry and capnography (P_ET_CO_2_). An indwelling cannula was then inserted into a large vein in the forearm for induction of general anesthesia and a second indwelling cannula was inserted into the contralateral radial artery for the continuous measurement of arterial blood pressure and for the collection of blood samples. Anesthesia was induced with intravenous midazolam 0.1-0.2 mg/kg, fentanyl 3.0-5.0 μg/kg, propofol 2–3 mg/kg and mivacurium (LOT: H20080520, Jiangsu Nhwa Pharmaceutical Co., LTD. Dosage and injection time was according to the subgroups). After intubation, anesthesia was maintained with a continuous infusion of propofol 50–100 μg/kg/min and remifentanil 0.3-0.5 μg/kg/min. Ventilation was adjusted to maintain P_ET_CO_2_ between 30–40 mmHg. Propofol and remifentanil were discontinued 5–10 min prior to the end of the operation.

### Measurements

#### Efficacy indicators

The primary outcome measurements of this clinical experiment were neuromuscular effects: (1) Times of T_1_ maximal depression and recovery to 5%, 25%, 50%, 75%, 95% were recorded; (2) The onset time (time from mivacurium administration to maximal depression of T_1_); (3) duration of clinical action (time from mivacurium administration to 25% recovery of T_1_); (4) recovery index (recovery of T_1_ from 25 to 75%), and return of T_4_/T_1_ (TOFR) to 90% were determined, respectively.

The secondary efficacy indicator was tracheal intubation conditions, which were evaluated according to the position of the vocal cords and airway reaction, using the evaluation scale of 1, 2, 3, and 4 which means excellent, good, poor or unable to intubate. Tracheal intubation was performed at maximal depression of T_1_ and carried out by an experienced anesthesiologist who was not told which agent was used.

#### Safety indicators

The primary safety indicator was plasma histamine concentration. Radial artery blood (1 ml) was sampled to quantify plasma histamine concentrations before mivacurium administration and 1, 4, and 7 min after mivacurium injection (P_0_, P_1_, P_2_ and P_3_). Samples were transferred to tubes containing potassium EDTA, kept on ice, and centrifuged at 3500 rpm at 4 °C for 10 min. After centrifugation, the plasma samples were stored at −80 °C until further analysis. Plasma concentrations of histamine were determined using a human histamine enzyme-linked immunosorbent assay (ELISA) kit.

The secondary safety indicator was anaphylaxis score (0 means no anaphylaxis; 1 means patients had signs of skin rash, but did not need medication; 2 means patient had obvious signs of skin rash and erythema, but did not need medication; 3 means airway pressure was increased or patient had bronchospasm, and needed medication), and other adverse effects (including bronchospasm, shock, etc.).

In addition, vital signs have been monitored all the way, such as BP, HR, ECG, SpO_2_. The change of BP and HR fluctuated within 30% of the baseline values. If beyond this range, the vasoactive agents would be given to treatment.

#### Statistical analysis

The data is presented as mean ± SD or numbers, as appropriate. The normal distribution of data was examined with the Kolmogorov-Smirnov test. Chi-square test was used for comparison of gender, grade of tracheal intubation conditions and the anaphylaxis score among groups. Differences in onset and recovery time between the groups were analyzed with the analysis of variance and post hoc tests (Least Significant Difference, LSD). Plasma histamine concentrations data was compared using repeated measures one-way analysis of variance and post hoc tests (LSD). Data was analyzed using SPSS software (SPSS 16.0, SPSS, Inc., Chicago, IL, USA). Unless otherwise specified, *P* < 0.05 was considered statistically significant.

## Results

**Fig. 1:**
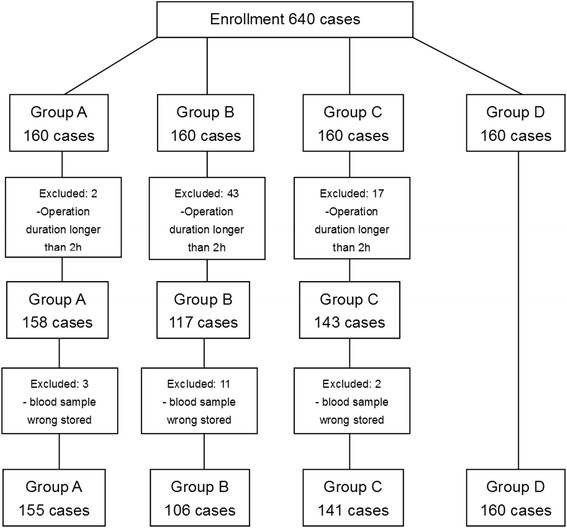
Flow chart of patient’s enrollment

The original data set included 640 patients. 62 patients were excluded owing to the operation duration was longer than 2 h; another 16 patients were excluded because their blood samples for plasma histamine concentration measurement were wrongly stored. In total, 562 cases completed the trial, as shown in Fig. 1.

The demographic data is summarized in Tables 1, 2, 3 and 4. The performed surgeries included 276 ENT cases, 135 orthopedic cases, and 151 urological cases. There were no sex ratio and body weight differences among the four subgroups in each group.

The data for grade of tracheal intubation conditions, cases of anaphylaxis score, and duration of surgery are shown in Tables 5, 6, 7 and 8. The excellent ratings of intubation conditions, which were described as grade 1, were from 74 to 100% among the 16 subgroups; when combined together with grade 1 and 2, the excellent ratings were 100% among 15 subgroups, except subgroup 14 in which 2.6% were considered as poor. The ratings of anaphylaxis score 0 were from 82 to 100% among the 16 subgroups. Two cases in each of subgroup 3 and 14, and one case in each of subgroup 7, 9 and 11 caused bronchospasm. After medication intervention, serious adverse effects were not found in this study.

**Table 5:** Patients’ data of group A as mean ± SD or numbers (*n* = 155)

Subgroups	n	Cases of the grade of tracheal intubation conditions (1/2/3/4)	Cases of the anaphylaxis score (0/1/2/3)	Duration of surgery (min)
Group 1	39	29/10/0/0	32/7/0/0	69 ± 34
Group 2	37	29/8/0/0	35/2/0/0	75 ± 40
Group 3	39	37/2/0/0	35/2/2/0	76 ± 40
Group 4	40	31/9/0/0	38/2/0/0	67 ± 33

There was no significant different among four subgroups

**Table 6:** Patients’ data of group B as Mean ± SD or numbers (*n* = 106)

Subgroups	n	Cases of the grade of tracheal intubation conditions (1/2/3/4)	Cases of the anaphylaxis score (0/1/2/3)	Duration of surgery (min)
Group 5	29	27/2/0/0	29/0/0/0	60 ± 22
Group 6	25	22/3/0/0	24/1/0/0	63 ± 32
Group 7	28	24/4/0/0	23/4/1/0	59 ± 21
Group 8	24	24/0/0/0	24/0/0/0	52 ± 28

There was no significant different among four subgroups

**Table 7:** Patients’ data of group C as mean ± SD or numbers (*n* = 141)

Subgroups	n	Cases of the grade of tracheal intubation conditions (1/2/3/4)	Cases of the anaphylaxis score (0/1/2/3)	Duration of surgery (min)
Group 9	34	27/7/0/0	32/1/1/0	56 ± 30
Group 10	36	35/1/0/0	35/1/0/0	50 ± 27
Group 11	35	32/3/0/0	34/0/1/0	54 ± 26
Group 12	36	30/6/0/0	32/4/0/0	53 ± 24

There was no significant different among four subgroups

**Table 8:** Patients’ data of group D as mean ± SD or numbers (*n* = 160)

Subgroups	n	Cases of the grade of tracheal intubation conditions (1/2/3/4)	Cases of the anaphylaxis score (0/1/2/3)	Duration of surgery (min)
Group 13	40	34/6/0/0	36/4/0/0	61 ± 27
Group 14	40	32/7/1/0	35/3/2/0	58 ± 26
Group 15	40	38/2/0/0	39/1/0/0	60 ± 32
Group 16	40	34/6/0/0	37/3/0/0	53 ± 21

There was no significant different among four subgroups

Tables 9, 10, 11 and 12 summarize the neuromuscular effects of mivacurium in four groups. In Group A, the onset time of Group 3 was shorter than that of Group 1 (189 ± 64 s vs. 220 ± 73 s, *P* = 0.043), and Group 4 was shorter than Group 2 (181 ± 60 s vs. 213 ± 71 s, *P* = 0.042); the recovery times were no statistical differences, as shown in Table 9. In Group C, the neuromuscular effects of mivacurium in the four subgroups were similar with respect to onset time and recovery index; the T1 25% recovery time of Group 9 was shorter than that of Group 11 (693 ± 188 s vs. 800 ± 206 s, *P* = 0.017), as shown in Table 11. The onset and recovery times of mivacurium were not statistically different in Group B and Group D, as shown in Tables 10 and 12.

**Table 9:** Neromuscular effects of mivacurium in paediatric patients in group A (mean ± SD, S)

Subgroups	n	Onset time	T1 5% recovery	T1 25% recovery	Recovery index	TOF ratio 90%
Group 1	39	220 ± 73	327 ± 95	585 ± 171	309 ± 96	893 ± 268
Group 2	37	213 ± 71^&^	308 ± 86	569 ± 180	297 ± 74	917 ± 230
Group 3	39	189 ± 64^*^	347 ± 112	659 ± 194	339 ± 112	975 ± 231
Group 4	40	181 ± 60	346 ± 97	603 ± 191	329 ± 110	951 ± 262

Group 1 vs. Group 3, ^*^ *P* = 0.043; Group 2 vs. Group 4, ^&^ *P* = 0.042

**Table 10:** Neromuscular effects of mivacurium in paediatric patients in group B (mean ± SD, S)

Subgroups	n	Onset time	T1 5% recovery	T1 25% recovery	Recovery index	TOF ratio 90%
Group 5	29	173 ± 51	310 ± 104	754 ± 138	307 ± 89	971 ± 171
Group 6	25	180 ± 56	295 ± 95	810 ± 150	314 ± 78	899 ± 215
Group 7	28	184 ± 60	300 ± 95	805 ± 179	296 ± 92	979 ± 280
Group 8	24	167 ± 52	303 ± 75	769 ± 161	287 ± 89	951 ± 206

There was no significant different among four subgroups

**Table 11:** Neromuscular effects of mivacurium in paediatric patients in group C (mean ± SD, S)

Subgroups	n	Onset time	T1 5% recovery	T1 25% recovery	Recovery index	TOF ratio 90%
Group 9	34	183 ± 57	263 ± 80	693 ± 188	313 ± 102	1014 ± 242
Group 10	36	172 ± 55	277 ± 89	741 ± 185	311 ± 91	1033 ± 215
Group 11	35	161 ± 51	296 ± 95	800 ± 206^#^	315 ± 94	901 ± 259
Group 12	36	160 ± 50	313 ± 96	743 ± 172	293 ± 91	958 ± 255

Group 9 vs. Group 11, ^#^ *P* = 0.017

**Table 12:** Neromuscular effects of mivacurium in paediatric patients in group D (mean ± SD, S)

Subgroups	n	Onset time	T1 5% recovery	T1 25% recovery	Recovery index	TOF ratio 90%
Group 13	40	184 ± 57	376 ± 116	796 ± 207	299 ± 80	911 ± 214
Group 14	40	187 ± 66	389 ± 116	796 ± 199	304 ± 94	998 ± 239
Group 15	40	181 ± 58	369 ± 95	789 ± 193	289 ± 79	974 ± 251
Group 16	40	182 ± 56	363 ± 116	794 ± 196	296 ± 95	963 ± 252

There was no significant different among four subgroups

For the age-corrected T_1_ 5% recovery time of 0.2 mg/kg mivacurium with 20 s injecting time, it was shorter in the 3–6 years aged patients (Group 3 vs. Group 9, *P* = 0.001). For the age-corrected T_1_ 25% recovery time of 0.2 mg/kg mivacurium with 20 s injecting time, it was longer in the 7–14 years aged patients (Group 3 vs. Group 13, *P* = 0.003). For the age-corrected T_1_ 5% recovery time of 0.2 mg/kg mivacurium with 40 s injecting time, it was shorter in the 3–6 years aged patients (Group 4 vs. Group 10, *P* = 0.003). For the age-corrected T_1_ 25% recovery time of 0.2 mg/kg mivacurium with 40 s injecting time, it was shorter in the 2–12 months yrs aged patients (Group 4 vs. Group 6, *P* = 0.000). For the age-corrected T_1_ 5% recovery time of 0.25 mg/kg mivacurium with 20 s injecting time, it was longer in the 7–14 years aged patients (Group 11 vs. Group 15, *P* = 0.001).

**Fig. 2:**
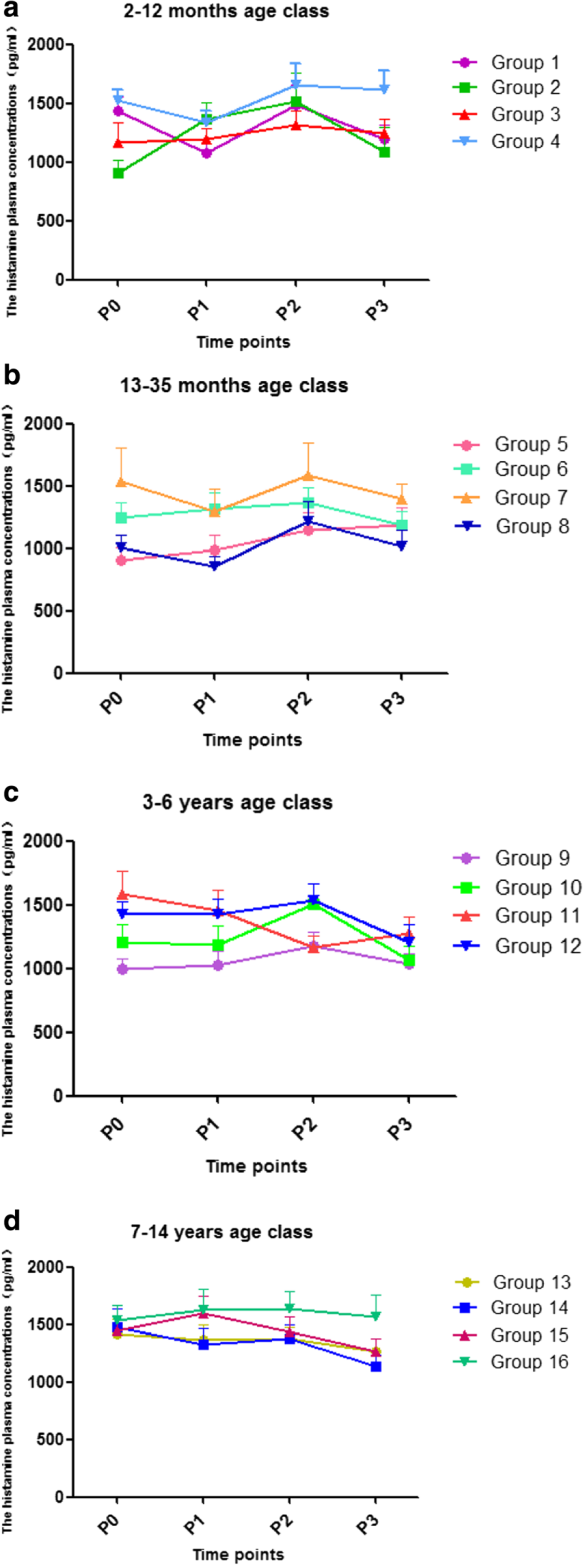
The plasma concentrations of histamine. The plasma concentrations of histamine at P0, P1, P2 and P3 time points in four age classes (Groups **a**, **b**, **c**, **d**). Compared with P0, the values of P1, P2 and P3 were not different among four subgroups in each age class

The plasma concentrations of histamine at P0, P1, P2 and P3 were not different among four subgroups in each age class (Fig. 2).

All children demonstrated complete spontaneous recovery of neuromuscular function. Compared with baseline values, the changes of blood pressure and heart rate were not different in all children.

## Discussion

This multimember trial was designed to evaluate onset and recovery time, and safety of mivacurium administered as a bolus in different aged children. Our results demonstrated that the onset time of 0.2 mg/kg mivacurium is shorter than 0.15 mg/kg mivacurium in the 2–12 months age class. This showed that increasing the dose of mivacurium accelerated the onset of neuromuscular block (Onset time: Group 3 vs. Group 1, 189 ± 64 s vs. 220 ± 73 s, *P* = 0.043; Group 4 vs. Group 2, 181 ± 60 s vs. 213 ± 71 s, *P* = 0.042), but did not prolong recovery time (TOF ratio 90%: 975 ± 231 s vs. 893 ± 268 s, *P* > 0.05; 951 ± 262 s vs. 917 ± 230 s, *P* > 0.05) in the 2–12 month age class. The T1 25% recovery time of 0.25 mg/kg mivacurium group was significantly longer than 0.2 mg/kg mivacurium group (T1 25% recovery time: Group 11 vs. Group 9, 800 ± 206 s vs. 693 ± 188 s, *P* = 0.017) in the 3–6 years age class. We also demonstrated that the induction dose of mivacurium and injection time do not affect on neuromuscular effects of mivacurium in 13–35 months and 7–14 years old children with respect to onset time, T1 5% recovery, T1 25% recovery, recovery index, and TOF ratio 90%. Shorten [7] reported that increasing the dose of mivacurium from 0.2 to 0.3 mg/kg accelerated the onset of block (time to 90% block, 1.6 ± 0.2 vs. 1.2 ± 0.2 min) (*P* < 0.001), but did not significantly prolong recovery (time to 95% recovery, 16.0 ± 3.8 vs. 18.6 ± 3.6 min) in 48 children aged 3–10 year. A further increase in dose to 0.4 mg/kg produced no significant decrement in onset time, but did prolong recovery (time to 95% recovery, 23.8 ± 5.0 min) (*P* < 0.001).

The age-corrected T1 5 and T1 25% recovery times were different in the four age classes with different induction doses of mivacurium and injection time. This difference among children is often explained by an age-dependent variation in volume of distribution and in differences in plasma cholinesterase activity [8]. Some studies in children have indicated that pChe is low at birth, increases to a maximum at the age of 3–6 years, and decreases to adult level at puberty [9]. Similarly, the present study showed that there was not a significant age-related increase of the onset time, recovery index, TOF ratio 90% among the four age classes.

Technique of measurement is also a crucial factor for determination of onset and recovery time [10]. We started our time measurements at the beginning of the injection of mivacurium, while most investigators started time measurements at the end of the injection of mivacurium.

Clinically significant histamine release may occur, especially in an adult patient [11]. Release of histamine is related to the dose and speed of injection [12]. In our study, these data suggested that mivacurium had no significant histamine release within the present dose range (the dose of mivacurium from 0.15 to 0.25 mg/kg), and mivacurium did not significantly change cardiocirculatory parameters. None of the serious adverse events were seen to be related to mivacurium. However, due to children’s immature cardiovascular and respiratory systems, adverse events associated with histamine release from mivacurium pose a greater risk to pediatric patients [13].

Because volatile anesthetics may have prolonged recovery independent from the neuromuscular block [14], we specifically avoided volatile anesthetics in our study. Remifentanil has been used successfully in anesthesia without muscle relaxant and maintained a stable hemodynamic state during non-muscle-relaxant anesthesia [15], so we choose it as the opioid agent.

## Conclusions

The induction dose and injection time of mivacurium had mostly insignificant effects on onset and recovery times. The main exception to this was that in 2–12 m aged patients, increasing the dose of mivacurium from 0.15 to 0.2 mg/kg accelerated the onset time by about 30 s. Mivacurium produced no significant release of histamine in any age group at the doses studied, and there were no serious adverse effects in all children.

## Abbreviations

ASA: American Society of Anesthesiologists
BP: Blood pressure
ECG: Electrocardiogram
ELISA: Enzyme-linked immunosorbent assay
HR: Heart rate
PACU: Post-anesthesia care unit
pChe: Plasma cholinesterase
PetCO_2_: End tidal carbon dioxide pressure
SpO_2_: Pulse oximetry
TOF: Train-of four stimulation
